# Author Correction: Breakdown of the scaling relation of anomalous Hall effect in Kondo lattice ferromagnet USbTe

**DOI:** 10.1038/s41467-024-55351-2

**Published:** 2024-12-20

**Authors:** Hasan Siddiquee, Christopher Broyles, Erica Kotta, Shouzheng Liu, Shiyu Peng, Tai Kong, Byungkyun Kang, Qiang Zhu, Yongbin Lee, Liqin Ke, Hongming Weng, Jonathan D. Denlinger, L. Andrew Wray, Sheng Ran

**Affiliations:** 1 Department of Physics, Washington University in St. Louis, St. Louis, MO 63130 USA; 2 Department of Physics, New York University, New York, NY 10003 USA; 3 Beijing National Laboratory for Condensed Matter Physics, Institute of Physics, Chinese Academy of Sciences, Beijing, 100190 China; 4 Department of Physics, University of Arizona, Tucson, AZ 85721 USA; 5 University of Nevada, Las Vegas, NV 89154 USA; 6 Ames lab, Ames, IA 50011 USA; 7 Advanced Light Source, Lawrence Berkeley National Laboratory, Berkeley, CA 94720 USA

Correction to: *Nature Communications* 10.1038/s41467-023-36221-9, published online 01 February 2023

The original version of this Article omitted relevant details in fifth sentence of the Acknowledgements. The incorrect version of the sentence in the Acknowledgements read:

“LQSGW and DMFT calculations have been carried out using resources of the National Energy Research Scientific Computing Center (NERSC), a US Department of Energy Office of Science User Facility operated under Contract No. DE-SC0021970.”

The correct version should read:

“LQSGW and DMFT calculations have been carried out using resources of the National Energy Research Scientific Computing Center (NERSC), supported by the Office of Science of the U.S. Department of Energy under Contract No. DE-AC02-05CH11231 using NERSC award BES-ERCAP0024866.”

This has now been corrected in both the PDF and HTML versions of the Article.

