# Supplementary material for: Breakdown of the scaling relation of anomalous Hall effect in Kondo lattice ferromagnet USbTe
Source: Nat Commun. 2023 Feb 1;14:527. doi: 10.1038/s41467-023-36221-9 (PMC9889341; doi:10.1038/s41467-023-36221-9)
Supplement: Supplementary file 1 — Supplementary Information [file 41467_2023_36221_MOESM1_ESM.pdf]

# Supplementary Information for
# Breakdown of the scaling relation of anomalous Hall effect in Kondo lattice ferromagnet USbTe

Hasan Siddiquee[1], Christopher Broyles[1], Erica Kotta[2], Shouzheng Liu[2], Shiyu Peng[3],Tai Kong[4], Byungkyun Kang[5], Qiang Zhu[5], Yongbin Lee[6], Liqin Ke[6], Hongming Weng[3], Jonathan D. Denlinger[7], L. Andrew Wray[2], Sheng Ran[1]
[1] *Department of Physics, Washington University in St. Louis, St. Louis, MO 63130, USA*
[2] *Department of Physics, New York University, New York, New York 10003, USA*
[3] *Beijing National Laboratory for Condensed Matter Physics, Institute of Physics, Chinese Academy of Sciences, Beijing 100190, China*
[4] *Department of Physics, University of Arizona, Tucson, AZ 85721, USA*
[5] *University of Nevada, Las Vegas, Nevada 89154, USA*
[6] *Ames lab, Ames, IA 50011, USA*
[7] *Advanced Light Source, Lawrence Berkeley National Laboratory, Berkeley, California 94720, USA*
(Dated: January 3, 2023)

## I. SUPPLEMENTARY NOTE 1: NORMAL HALL EFFECT

Five probe transport measurements were performed for longitudinal and Hall resistivity simultaneously. To correct for contact misalignment, the measured longitudinal and Hall resistivity was field-symmetrized and field-antisymmetrized, respectively. Using slope of linearly decreased portion of the corrected Hall resistivity we calculated normal Hall resistivity. Subtracting normal Hall resistivity contribution from corrected Hall resistivity we obtained anomalous Hall resistivity, as shown in Supplementary Fig. 1.

## II. SUPPLEMENTARY NOTE 2: ANALYSIS OF AHE OF SRRUO$_3$

As our anomalous Hall data violates the conventional scaling relation for a large temperature range, we need to pay careful attention to the way how we analyze our data. Particularly, the equation we use in the main text $\rho_{xy}^a = a(M)\rho_{xx} + b(M)\rho_{xx}^2$ seems to depend on the $M$ dependence of $b(M)$. Indeed, some materials exhibiting AHE due to the intrinsic mechanism shows nonlinear $M$ dependence of $b(M)$. A well known example is $SrRuO_3$ [1], in which both experimental data and calculations show that the intrinsic AHE has nonlinear dependence on $M$. However, our analysis focus on two temperature range in which either $M$ is a constant or intrinsic mechanism vanishes. When intrinsic contribution vanishes, $\rho_{xy}^a = a(M)\rho_{xx} + b(M)\rho_{xx}^2$ reduces to $\rho_{xy}^a = a(M)\rho_{xx}$, no $b(M)$ term. When $M$ reaches a constant value at low temperature, $\rho_{xy}^a = a(M)\rho_{xx} + b(M)\rho_{xx}^2$ reduces to $\rho_{xy}^a = C_1\rho_{xx} + C_2\rho_{xx}^2$, no $M$ dependence either. To further illustrate that this analysis is valid when $M$ reaches constant value, we analyzed the published data of $SrRuO_3$ in the same way as our Fig. 2e in the main text, shown in Supplementary Fig. 2. It is clear that, AHE of $SrRuO_3$ follows a expected behavior of the scaling relation, a linear line, below about 50 K, where $M$ is constant. One can use the slope and intersect to calculate the intrinsic and skew scattering contribution to AHE. This example demonstrate that even when intrinsic contribution to AHE has strong nonlinear $M$ dependence, the scaling relation we use is valid in the temperature range where $M$ is constant.

## III. SUPPLEMENTARY NOTE 3: KONDO COHERENCE IN MAGNETICALLY ORDERED STATE

Coherence between itinerant electrons and localized f-electron degrees of freedom provides a mechanism for the low temperature emergence of a f-electron band with partial-integer spectral weight within a few millielectron volts of the Fermi level, even when the bare $f$-electron energies and DFT bands are far removed in energy [2–4]. This phenomenon is best known in the context of the Kondo singlet, but can be thought of more broadly as a consequence of near-degeneracy in the base atomic multiplet manifold of an $f$-electron system [4]. The low crystal field symmetry of the SbTe cage around uranium provides a mechanism to preserve the near-degeneracy of low energy crystal field-split states beneath a high temperature magnetic transition [4], and is associated with the coexistence of $T_N \sim 200$ K magnetism with lower temperature ($T <$ 100 K) many-body coherence phenomena in closely related $USb_2$ [5–7].

## IV. SUPPLEMENTARY NOTE 4: ARPES INCIDENT ENERGY

Incident energy dependence is shown in Supplementary Fig. 3, revealing significant two-dimensional character near the Fermi level. The inner potential is not determined with full confidence, and we note that the presence of two uranium layers per unit cell of the crystal is expected to yield $k_z$ resonances that do not match the $2\pi/c$ dispersion periodicity.

## V. SUPPLEMENTARY NOTE 5: BAND STRUCTURE AND ANOMALOUS HALL CONDUCTIVITY BASED ON DFT CALCULATIONS

The results of the DFT calculations show that the bands near the Fermi surface mainly consist of $f$-orbital of U atoms, as shown in Supplementary Fig. 4a. We want to point some discrepencies between the GGA+U calculations and ARPES/DMFT calculations. The GGA+U calculations in Supplementary Fig. 3 includes the $\overline{Z}$-$\overline{G}$ high symmetry axis, from which we can see that about 1/2 of the bands surrounding the Fermi level have significant $z$-axis dispersion. The DMFT spectral function in Fig. 4a of the main text looks quite different, with a far more 2D character for bands just beneath the Fermi level. Correlations considered by DMFT bring this about in several ways such as: (1) by bringing relatively non-dispersive and quasi-2D $f$-states to the Fermi level; (2) by splitting each $5f$ band into multiple bands; (3) split bands could have downward-renormalized creation/annihilation operators and thus reduced dispersion (and reduced spectral weight). All three of these effects cause the observed band structure to be more two-dimensional. However they do not eliminate the expectation that there will be some bands with significant 3D character. The ARPES measurement in Supplementary Fig. 3 shows some quasi-2D features, but also shows a number of features that can not be confidently traced through the Brillouin zone due to strong matrix element effects. These matrix element effects are in part associated with interference with the near-periodic substructures of the unit cell. For example, one expects a beat frequency of $2\pi/d$, where $d$ is the distance between the two uranium sublattices. Broadening along the $k_z$-axis is also very relevant, but there are no clear-cut indicators that it is a dominant influence in this case, and it can be difficult to assess by eye for a complex band structure.

From the obtained electronic structures, it's easy to acquire anomalous Hall conductivity (AHC) by integrating directly the Berry curvature of the occupied states, as depicted in Supplementary Fig. 4b. The calculated AHC value turns negative when the chemical potential shifts upwards above the Fermi level, and reaches its global minimum at about 0.5 eV. With a chemical potential of $\sim 0.4$ eV, both the sign and magnitude of the calculated AHC matches our experimental data. At the first glance, this seems to indicate inconsistency between DFT calculations and experiment results. On the other hand, AHC highly depends on the $f$ bands around the Fermi level. The GGA+U method is very limited when treating the strongly correlated $f$ electrons, which tends to lead to incorrect Fermi level. It also does not account for the splitting of $f$ orbital multiplicities and their renormalization. Therefore the number of $f$ bands in DFT calculations is typically different from that obtained from ARPES measurements and DMFT calculations. Nevertheless, the dispersion of the $f$ bands at the Fermi level from ARPES/DMFT results resembles that of the $f$ bands between 0.3 and 0.5 eV in DFT calculations. This provides additional support that the observed intrinsic anomalous Hall effect is closely related to the emerged coherent $f$ bands.


[1] F. Zhong, N. Naoto, S. Takahashi Kei, A. Atsushi, M. Roland, O. Takeshi, Y. Hiroyuki, K. Masashi, T. Yoshinori, and T. Kiyoyuki, Science **302**, 92 (2003).

[2] F. Lu, J. Zhao, H. Weng, Z. Fang, and X. Dai, PRL **110**, 096401 (2013).

[3] M. Dzero, J. Xia, V. Galitski, and P. Coleman, Annu. Rev. Condens. Matter Phys. **7**, 249 (2016).

[4] P. Thunström and K. Held, PRB **104**, 075131 (2021).

[5] L. Miao, R. Basak, S. Ran, Y. Xu, E. Kotta, H. He, J. D. Denlinger, Y.-D. Chuang, Y. Zhao, Z. Xu, J. W. Lynn, J. R. Jeffries, S. R. Saha, I. Giannakis, P. Aynajian, C.-J. Kang, Y. Wang, G. Kotliar, N. P. Butch, and L. A. Wray, Nature Communications **10**, 644 (2019).

[6] I. Giannakis, J. Leshen, M. Kavai, S. Ran, C.-J. Kang, S. R. Saha, Y. Zhao, Z. Xu, J. W. Lynn, L. Miao, L. A. Wray, G. Kotliar, N. P. Butch, and P. Aynajian, Science Advances **5**, eaaw9061 (2022).

[7] Q. Y. Chen, X. B. Luo, D. H. Xie, M. L. Li, X. Y. Ji, R. Zhou, Y. B. Huang, W. Zhang, W. Feng, Y. Zhang, L. Huang, Q. Q. Hao, Q. Liu, X. G. Zhu, Y. Liu, P. Zhang, X. C. Lai, Q. Si, and S. Y. Tan, PRL **123**, 106402 (2019).

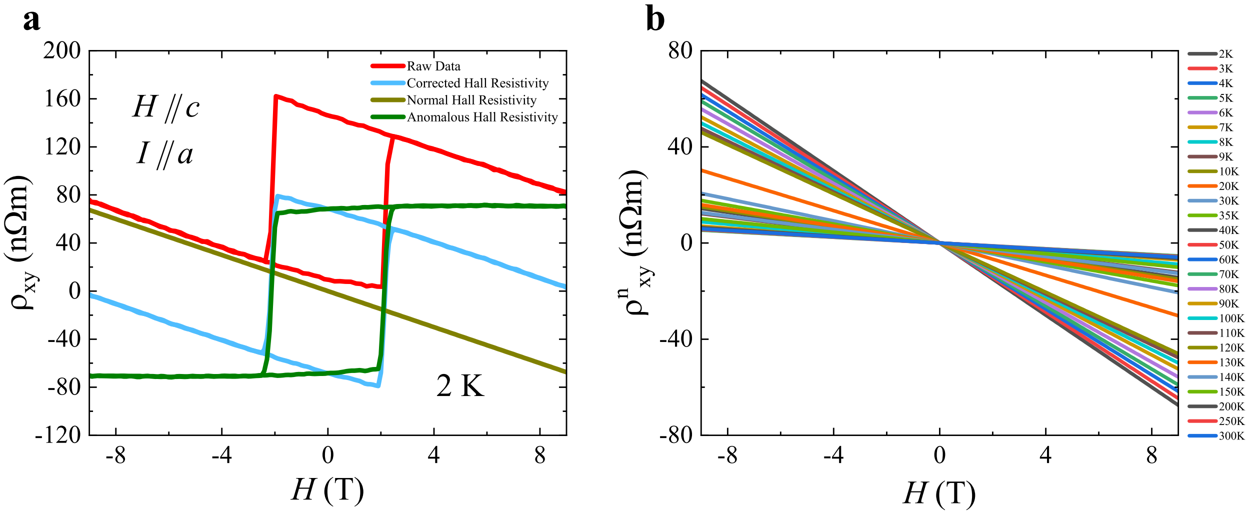


Supplementary Fig. 1. (a) Magnetic field dependence of the Hall resistivity $\rho_{xy}$ of USbTe single crystal at 2 K. (b) Temperature dependence of the normal Hall resistivity $\rho_{xy}^{n}$.

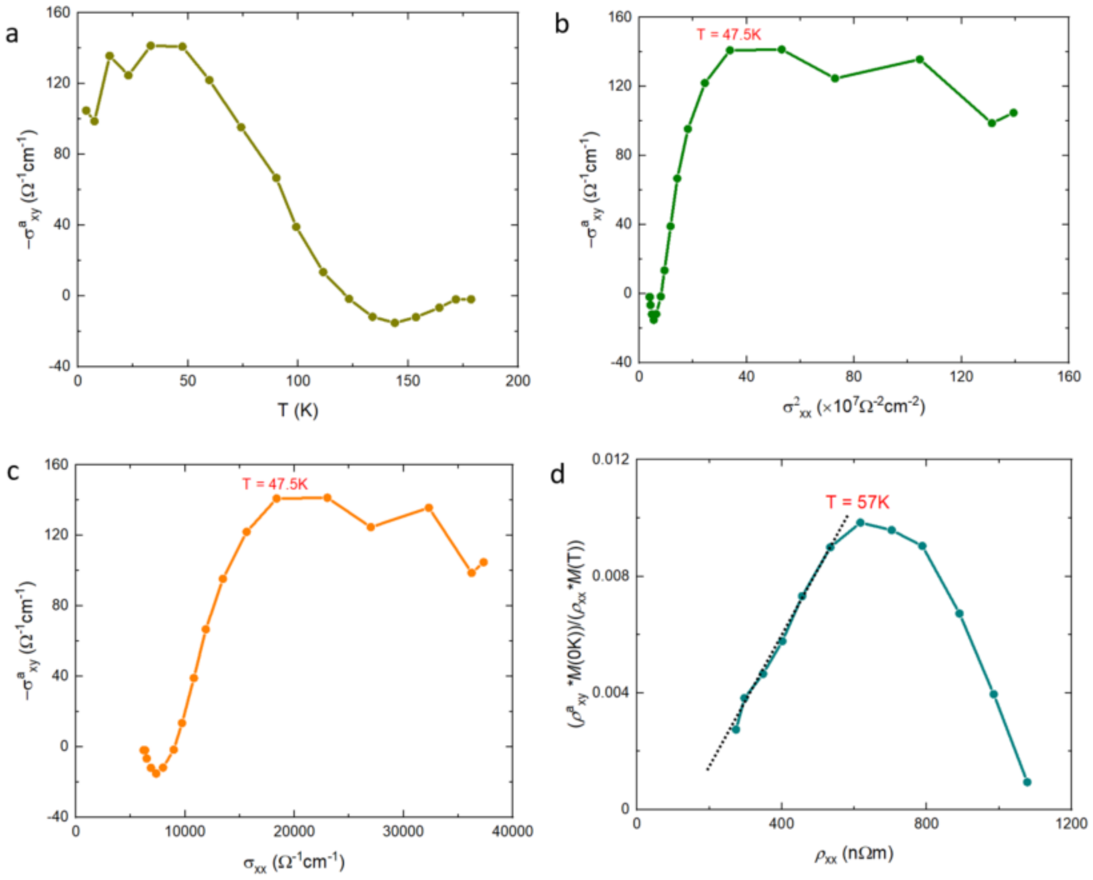


Supplementary Fig. 2. (a-d) AHE data of $SrRuO_3$ [1] plotted in the same way as we do in the main text Fig. 2d-f.

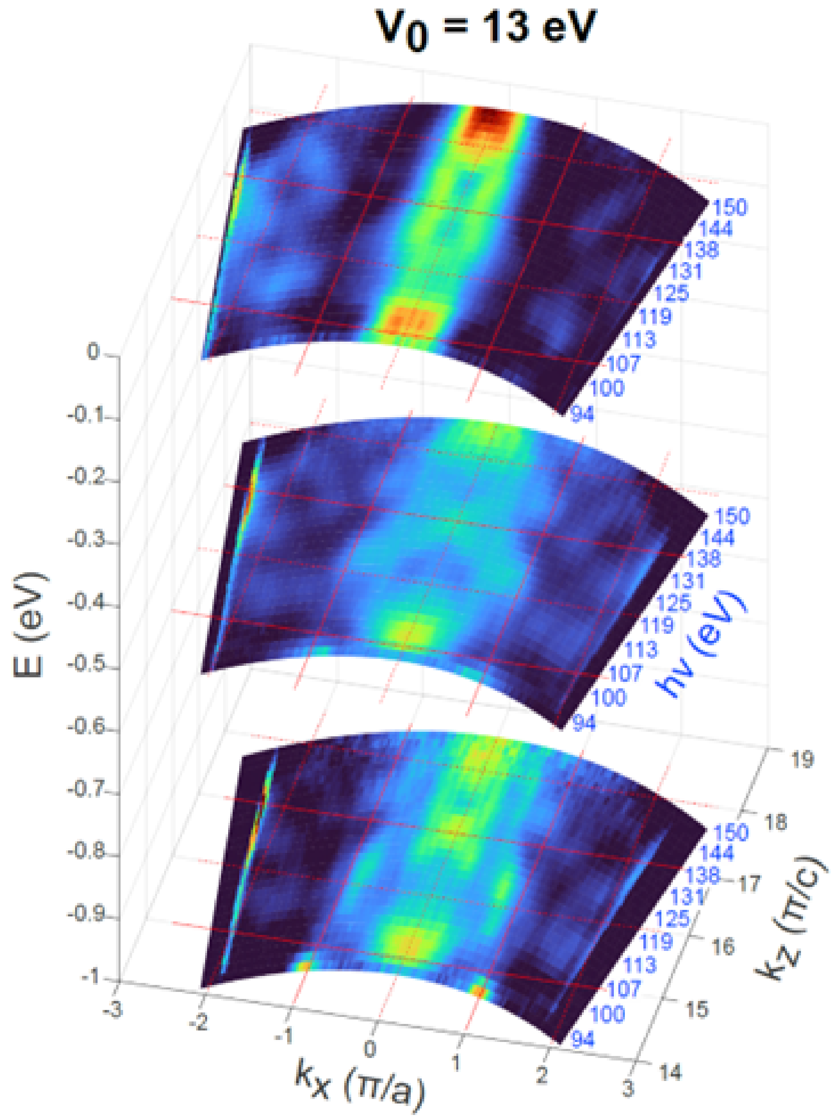


Supplementary Fig. 3. Incident energy dependence is shown at energies of $E = 0$, 0.5 and 1.0 eV relative to the Fermi level. Momentum along the $z$-axis is evaluated using a speculatively attributed inner potential of $V_0 = 13$ eV.

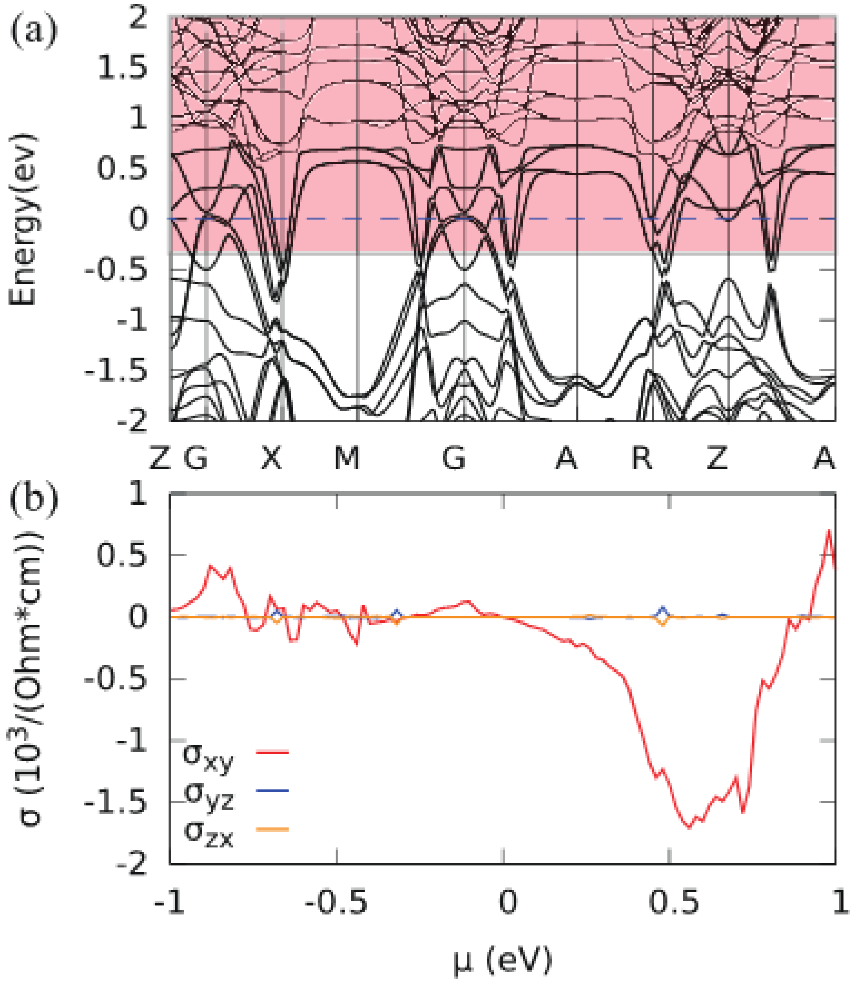


Supplementary Fig. 4. (a) Bulk band structure of GGA+FM+SOC (U = 4.0 eV, J = 0.57 eV). The bands in the shadow region consist mainly of $f$-orbital of U atoms. The blue dashed line indicates the Fermi level. (b) The anomalous Hall conductivity vs chemical potential ($\mu$). It shows that the only nontrivial component of anomalous Hall conductivity is $\sigma_{xy}$. It is negligible originally, but it turns negative when the chemical potential shifts upwards and reaches its global minimum at about 0.5 eV.
